# Magnetic resonance imaging in categorization of ovarian epithelial cancer and survival analysis with focus on apparent diffusion coefficient value: correlation with Ki-67 expression and serum cancer antigen-125 level

**DOI:** 10.1186/s13048-019-0534-0

**Published:** 2019-06-26

**Authors:** Guofu Zhang, Weigen Yao, Taotao Sun, Xuefen Liu, Peng Zhang, Jun Jin, Yu Bai, Keqin Hua, He Zhang

**Affiliations:** 1 Department of Radiology, Obstetrics and Gynecology Hospital, Fudan University, Shanghai, People’s Republic of China; 2 Department of Radiology, Yuyao People’s Hospital, Ningbo, Zhejiang province People’s Republic of China; 3 Department of Radiology, International Peace Maternity and Child Health Hospital, Shanghai Jiao Tong University School of Medicine, Shanghai, People’s Republic of China; 4 Department of Pathology, Obstetrics and Gynecology Hospital, Fudan University, Shanghai, People’s Republic of China; 5 Center for Child and Family Policy, Duke University, Durham, USA; 6 Department of Gynecology, Obstetrics and Gynecology Hospital, Fudan University, Shanghai, People’s Republic of China

**Keywords:** Ovarian epithelial cancer, Magnetic resonance imaging, Diffusion magnetic resonance imaging, Prognostic factor

## Abstract

**Background:**

To determine whether magnetic resonance (MR) imaging features combined with apparent diffusion coefficient (ADC**)** values could be used as a tool for categorizing ovarian epithelial cancer (OEC) and predicting survival, as well as correlating with laboratory tests (serum cancer antigen 125, serum CA-125) and tumor proliferative index (Ki-67 expression).

**Methods and materials:**

MRI examination was undertaken before invasive procedures. MRI features were interpreted and recorded on the picture archive communication system (PACS). ADC measurements were manually performed on post-process workstation. Clinical characteristics were individually retrieved and recorded through the hospital information system (HIS). Cox hazard model was used to estimate the effects of both clinical and MRI features on overall survival.

**Results:**

Both clinical and MRI features differed significantly between Type I and Type II cancer groups (*p* < 0.05). The mean ADC value was inversely correlated with Ki-67 expression in Type I cancer (*ρ* = − 0.14, *p* < 0.05). A higher mean ADC value was more likely to suggest Type I ovarian cancer (Odds Ratio (OR) = 16.80, *p* < 0.01). Old age and an advanced International Federation of Gynecology and Obstetrics (FIGO) stage were significantly related to Type II ovarian cancer (OR = 0.22/0.02, *p* < 0.05). An advanced FIGO stage, solid components, and old age were significantly associated with poor survival (Hazard Ratio (HR) = 23.54/3.69/2.46, *p* < 0.05). Clear cell cancer type had a poorer survival than any other pathological subtypes of ovarian cancer (HR = 13.6, *p <* 0.01).

**Conclusions:**

MR imaging features combined with ADC value are helpful in categorizing OEC. ADC values can reflect tumor proliferative ability. A solid mass may predict poor prognosis for OEC patients.

**Electronic supplementary material:**

The online version of this article (10.1186/s13048-019-0534-0) contains supplementary material, which is available to authorized users.

## Background

Ovarian cancer is the most lethal disease in women’s gynecological malignancies [1]. In China, the latest epidemiology survey disclosed that there were an estimated 52,100 newly diagnosed cases and 22,500 deaths in 2015, and the mortality has still been increasing over the past decade [2]. Ovarian epithelial cancer (OEC) accounts for more than 70% of all ovarian malignancies. Based on the histology and gene research, OEC is classified into two subtypes: Type I and Type II ovarian cancer. Type I cancer includes low-grade endometrioid, mucinous, clear cell, and serous carcinomas. Type II cancer mainly comprises of high-grade serous, endometrioid, undifferentiated carcinomas, and malignant mixed mesodermal tumors [3, 4]. Both Type I and Type II OEC subgroups have unique biological behaviors and treatment responses [5]. Therefore, how to trigger patients into the right category before invasive procedure will help clinicians to design individual therapy. Sonography is the first modality of choice in evaluating adnexal masses. However, for most of complicated cases, MR imaging is usually needed to determine tumor characteristics in most of tertiary medical centers [6]. In gynecological malignancies, an ADC value derived from diffusion weighted imaging (DWI) is generally lower than benign conditions [7–10]. Currently, CA-125 is considered an important biomarker for early detection and a prognostic tool for prediction of ovarian disease recurrence after treatment [11, 12]. Ki-67 antigen is a marker of cell proliferation and a higher Ki-67 expression is associated with more aggressive disease and worse survival rates in OEC patients [13, 14]. Owing to the advancement of imaging techniques, imaging biomarkers have gradually gained attention for their potential application [15]. It has been reported that MRI parameters could be used to predict survival in cancer patients [16]. Nakamura et al. reported that the ADC value was an independent prognostic factor for disease-free survival after radical hysterectomy in patients with cervical cancer [17]. Until now, there has been limited literature reported for categorization and survival analysis correlated with MRI features, especially with ADC measurements, for patients with both OEC subtypes. The purpose of this study was two folds: First, to clarify whether OEC MRI characteristics coupled with clinical markers (Ki-67 expression and serum CA-125 level) could be used as an index for differentiating between Type I and Type II OEC and second, to determine that some MRI features could be used as prognostic factors for predicting overall survival for OEC patients.

## Methods

### Patients

Our institutional review board (Gynecological and Obstetric Hospital, School of Medicine, Fudan University, P.R.China) approved this retrospective study, and the requirement for informed consent was waived for all participants. From January to December 2017, data from 438 patients who underwent MRI examination for clinically suspected gynecological disease were retrieved from our institutional patient archive communication system (PACS). The inclusion criteria included: 1) no previous pelvic surgery; 2) no previous gynecological disease history; and 3) MRI examinations before pelvic or laparoscopic surgery that was performed at our institution. The exclusion criteria were: 1) previous pelvic surgical history or radiation history; 2) MRI data was unavailable either as the examination was performed in another institution or from claustrophobia; and 3) no histological results. Finally, the total number of patients in the studied sample was 250 (average age, 52.7 ± 12.3 years). The sample comprised 145 patients with Type I (24 cases of low-grade serous carcinomas, 17 endometroid carcinomas, 31 clear cell carcinomas, and 74 borderline serous/mucinous cystadenomas (BOTs) and 105 patients with Type II (high-grade serous carcinoma, HGSC) cancer. All of the included samples were pathologically proved by laparoscopy or laparotomy. All patients were followed up every 6 months during the first 3 years, and annually thereafter.

### MR acquisition and interpretation

MRI was performed using a 1.5-T MR system (Magnetom Avanto, Siemens) with a phased-array coil. The routine MRI protocols used for the assessment of pelvic masses included the axial turbo spin-echo T1-weighted imaging (T1WI), sagittal TSE T2-weighted imaging (T2WI), and axial/sagittal fat-suppressed T2WI (FS T2WI). DWI was performed in the axial plane with parallel acquisition technique by using *b* value = 0, 100, and 800 s/mm^2^. The detailed MRI acquisition parameters are listed in Additional file 1: Table S1. All lesion interpretation was performed by one experienced radiologist (H.Z. with more than 10 years of experience). The location, size (the largest dimension in two orthogonal planes), irregular septa (present/absent), and mass component (mainly solid with more than 70% solid components, mainly cystic with more than 70% cystic components, and mixed components), visibility of high signal on T1WI (hemorrhage or mucinous protein signal) within the lesion, and presence of both pelvic-free fluid and lymph nodes were noted. The ADC value was calculated by one observer (X.L.) on a commercially available post- processing workstation (Leonardo, Siemens, Germany). Regions of interest were drawn manually in both the cystic and solid areas, with no more than three sites in each lesion on *b* = 800 mm^− 2^/s DWI images. A circle or ellipsis with an area range of 160–320 mm^2^ was placed centrally in the targeted region. Only the lowest ADC value was used for the subsequent statistical analysis. Clinical characteristics (age, FIGO stage, serologic CA-125 level before treatment, and Ki-67 expression) were respectively recorded through the hospital information system.

### Data statistical analysis

Continuous variables were expressed as the mean ± standard deviation. A logistic model was applied to assess the effects of MRI parameters and laboratory tests on ovarian cancer subgroups. A Cox hazard regression model was established for survival analysis. Missing data in this study accounted for 1.4 to 23.9% across the variables. Multiple imputation procedures were applied to handle missing data issue in this study. Stata 14.2 (StataCorp, Austin, TX, USA) was used to perform statistical analyses. *P* values < .05 were considered statistically significant.

## Results

### Comparison between patients with type I and type II OEC

**Fig. 1:**
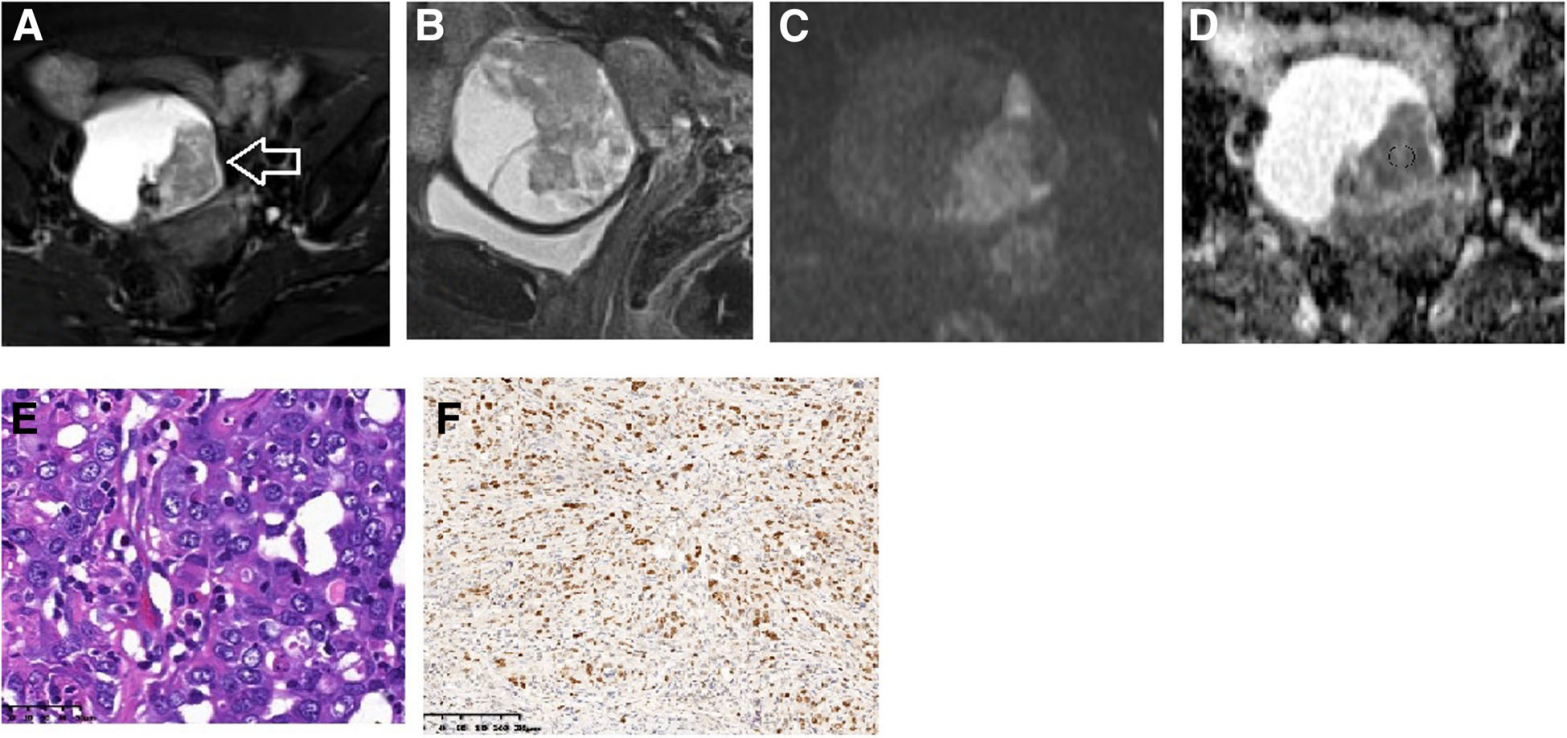
A 57-year-old woman with HGSC (IIIb). On axial fs-T2WI (**a**) and sagittal fs-T2WI (**b**), the mass showed as a mixed mass with intact capsule. On DWI (**c**), the solid tumor showed intermediate signal and the corresponding ADC value (**d**) was 729 (627–824) × 10^− 3^ s/m^2^. Hematoxylin and eosin staining of tumor (original magnification × 400, **e**) and Ki-67 stain picture (original magnification × 100, 70% expression in one view field, **f**)

**Fig. 2:**
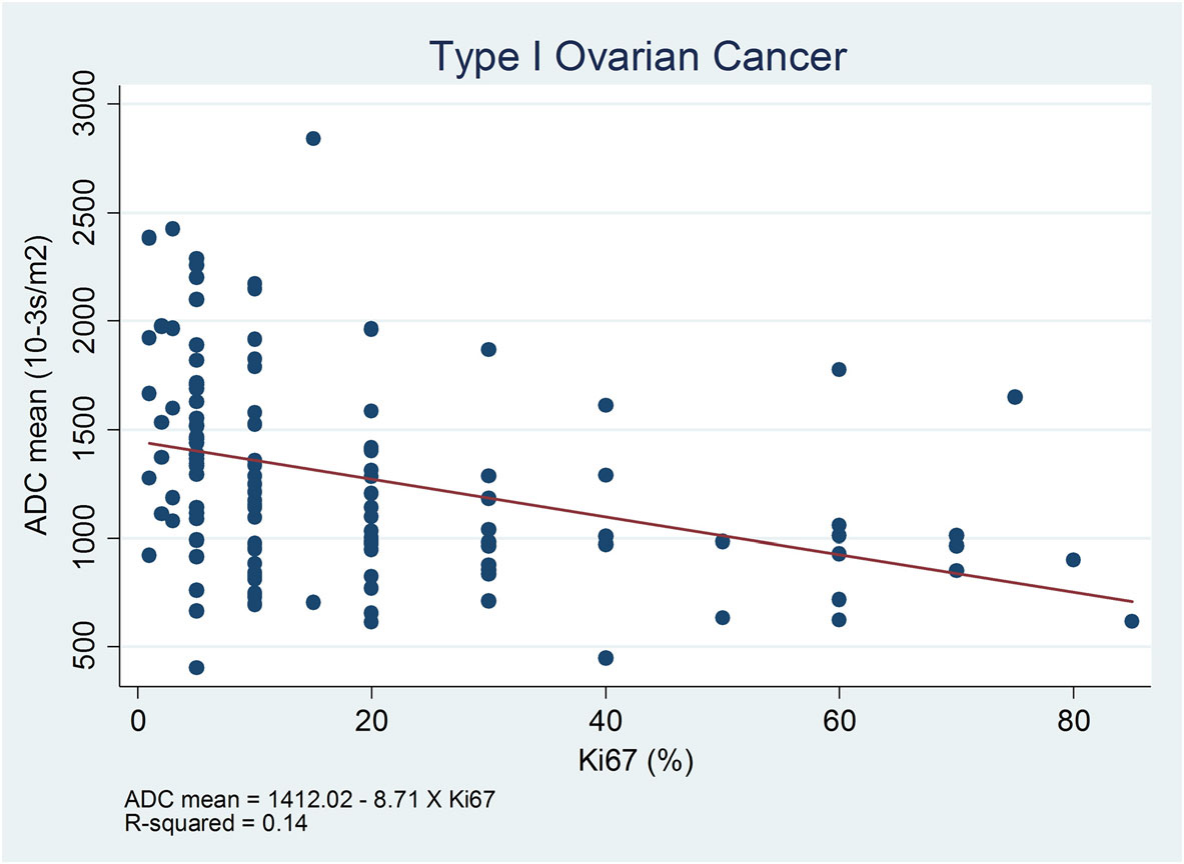
Scatter Plots of Ki-67 expression and the mean ADC value in Type I cancer group

Considering clinical parameters, Type II cancer (Fig. 1) was found in older patients (average 54.4 yrs.) and those with a more advanced FIGO stage (average 2.6) when compared with Type I cancer (average 44.0 yrs. and average FIGO stage 1.7, Additional file 1: Figure S1) (*p* < 0.01). The Ki-67 expression and CA-125 level in the Type II group were also higher than Type I cancer (36.5% vs. 18.0%, *p* < 0.01; 839.1 IU/mL vs. 220.0 IU/mL, *p* < 0.01, respectively). Statistically significant differences in MRI parameters were also noted between both groups. Type II OEC had a lower average ADC value (819.4 vs. 1275.9 × 10^− 3^ s/m^2^, *p* < 0.01; Additional file 1: Figure S2), a smaller tumor size (74.7 mm vs. 112.7 mm, *p* < 0.05), and less likelihood of presenting with septa and high signal on T1WI than Type I ovarian cancer (*p* < 0.05) (Table 1). The significant difference in the mean ADC value was also observed across both clinical and MRI subgroups (Additional file 1: Table S2). The ADC value was inversely related with Ki-67 expression in Type I cancer (*ρ* = − 0.14, *p* < 0.05) (Fig. 2), while it was not significantly related with the Type II cancer group (*ρ* = 0.02, *p* > 0.05) (Additional file 1: Figure S3).

**Table 1:** Summaries of detailed clinical and MRI characteristics of the studied population

	Type I	Type II	
	(*n* = 145)	(*n* = 105)	*p* value
Clinical features			
Age (yrs.)	43.3 ± 13.9	54.5 ± 9.9	0.00
< 30	27 (96.4%)	1 (3.6%)	
30–50	61 (66.3%)	31 (33.7%)	
> 50	57 (43.8%)	73 (56.2%)	
CA125 level (IU/mL)	206 ± 326	843 ± 1141	0.00
< 35	34 (75.6%)	11 (24.4%)	
35–200	49 (74.2%)	17 (25.8%)	
201–500	16 (44.4%)	20 (55.6%)	
> 500	16 (26.7%)	44 (73.3%)	
Ki67expression (%)	19 ± 20	36 ± 22	0.00
< 50	117 (63.6%)	67 (36.4%)	
50–75	12 (30.8%)	27 (69.2%)	
> 75	2 (22.2%)	7 (77.8%)	
FIGO Stage			0.00
I	59 (75.6%)	19 (24.4%)	
II	11 (52.4%)	10 (47.6%)	
III	26 (32.1%)	55 (67.9%)	
IV	1 (8.3%)	11 (91.7%)	
MRI features			
Maximum diameter (mm)	112.7 ± 4.9	74.7 ± 3.32	0.00
< 30	3 (42.9%)	4 (57.1%)	
30–50	12 (33.3%)	24 (66.7%)	
51–100	54 (52.9%)	48 (47.1%)	
> 100	71 (73.2%)	26 (26.8%)	
Component			0.00
Solid	21 (58.3%)	15 (41.7%)	
Cyst	20 (29.9%)	47 (70.1%)	
Mixed	99 (71.2%)	40 (28.8%)	
Mean ADC (SD.) × 10^−3^ s/m^2^	1275.9 (491.9)	819.4 (222.7)	0.00
Solid	1086(214)	814(187)	
Cyst	1015(517)	805(187)	
Mixed	1309(507)	856(242)	
Septa			0.01
Absent	72 (50.0%)	72 (50.0%)	
Present	68 (70.1%)	29 (29.9%)	
High signal on T_1_WI			0.00
Absent	75 (46.6%)	86 (53.4%)	
Present	65 (81.3%)	15 (18.7%)

### Clinical and MRI predictors of ovarian cancer subtypes

Older patients were less likely to be diagnosed with Type I (OR = 0.22, *p* < 0.01). In addition, patients in a more advanced stage were also less likely to be diagnosed with Type I (*p* < 0.05). Alternatively, if either a higher mean ADC value or a larger tumor was found on MRI, the patients would be more likely to be diagnosed with Type I ovarian cancer (OR = 16.80, *p* < 0.01; OR = 2.24, *p* < 0.01, respectively). When leaving BOTs out, a higher ADC value also predicted a Type I cancer diagnosis (Table 2).

**Table 2:** Multivariate logistic regression analysis of OEC subtypes based on clinical and MRI features

Type	OR	P	95% Confidential Interval (CI)	
Age	0.22	0.00	0.10	0.47
CA 125 level (IU/mL)				
< 35(reference)				
35–200	1.58	0.54	0.36	6.99
201–500	0.56	0.46	0.12	2.61
> 500	0.37	0.18	0.09	1.59
Ki-67 expression (%)				
< 50(reference)				
50–75	0.36	0.08	0.12	1.13
> 75	0.22	0.14	0.03	1.65
FIGO				
I (reference)				
II	0.39	0.17	0.10	1.50
III	0.14	0.00	0.05	0.44
IV	0.02	0.01	0.00	0.30
Maximum diameter (mm)	2.24	0.01	1.25	4.01
Component				
Mixed (reference)				
Cyst	0.62	0.42	0.20	1.97
Solid	0.84	0.77	0.27	2.65
mean ADC (Log)	16.80	0.00	4.20	67.20
Septa	0.48	0.16	0.17	1.34
High signal on T_1_WI	2.07	0.16	0.74	5.79

Note: Reference groups: ovarian cancer type II, component 1, absence of septa, and absence of hemorrhage. Multiple imputation approach was applied

### Survival analysis

**Fig. 3:**
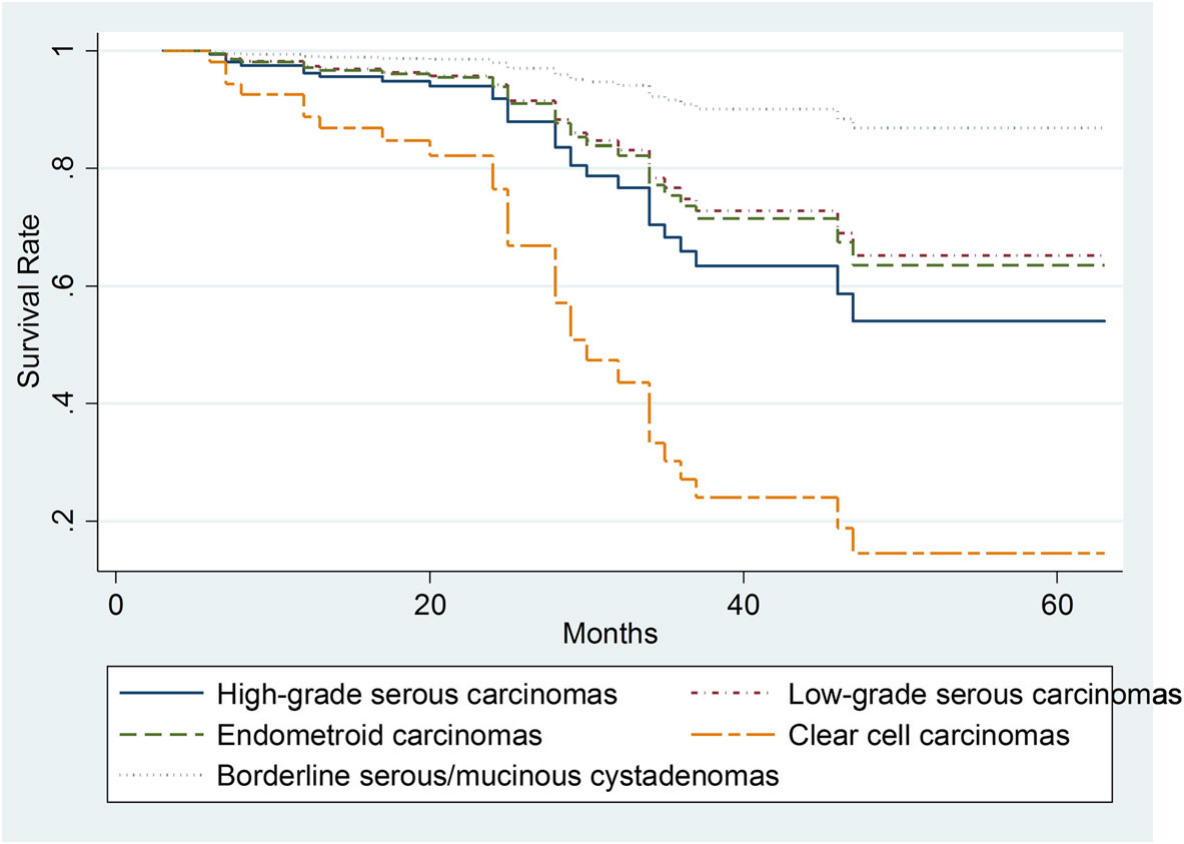
The Kaplan–Meier Plots of overall survival time for all follow-up patients based on different ovarian cancer pathological types

**Fig. 4:**
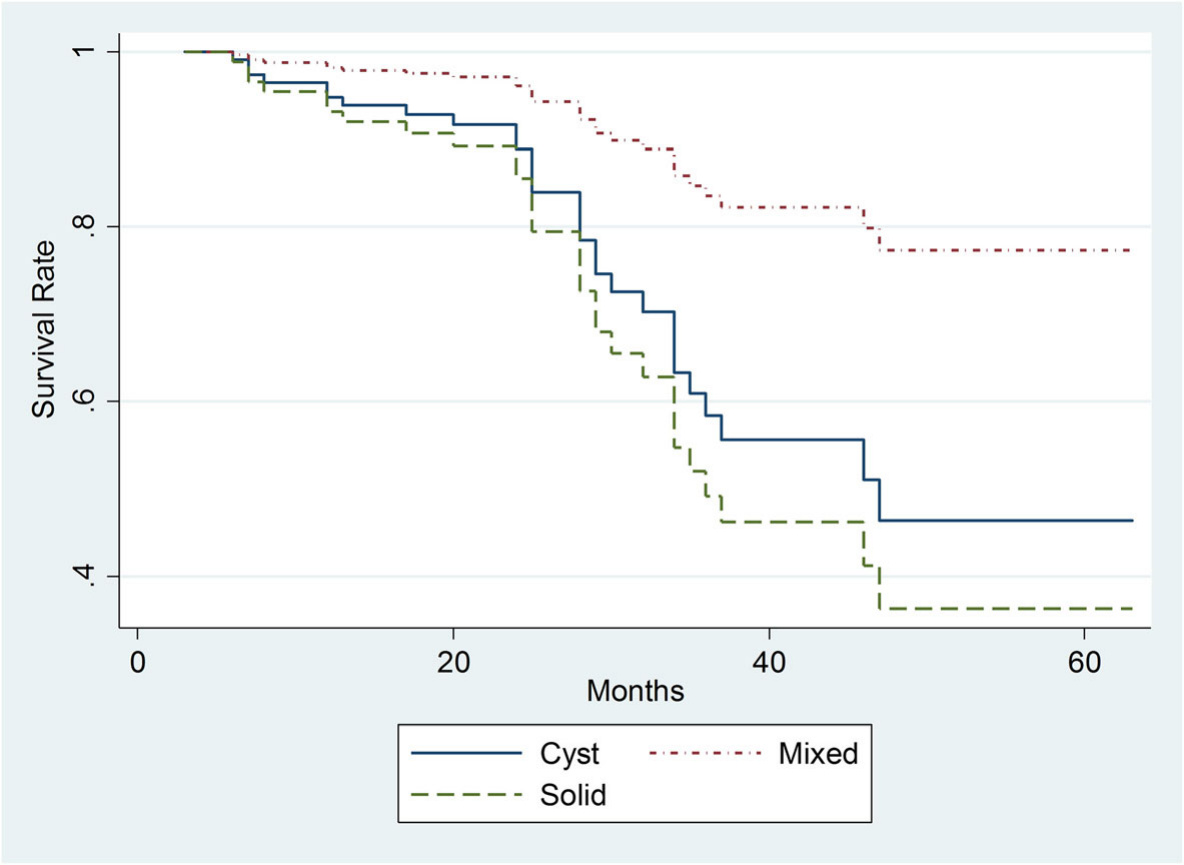
The Kaplan–Meier Plots of overall survival time for all follow-up patients based on mass component on MRI

Twenty-nine out of the 172 patients had died by the end of study (16.9%). Median durations of this studied group were about 27.2 months. The median follow-up period for the survivors was 27.6 months at the end time point (interquartile range, 18–35.5 months). Univariate analysis of overall survival showed that an older age, more advanced FIGO stage, solid component, and pathological subtypes were significant predictors of poor prognosis (*p* < 0.01). Specifically, clear cell cancer type had a poorer survival than borderline serous/mucinous cystadenomas (HR = 13.6, *p <* 0.01, Fig. 3). The survival time did not differ between Type I and Type II cancer types (Table 3). The FIGO stage is an independent prognostic factor and an advanced stage was related to death (*p* < 0.05). The solid mass and older age were more likely associated with a poor survival (HR = 3.69, *p* < 0.05, Fig. 4; HR = 2.46, *p* < 0.05). Neither OEC subtypes, ADC value, tumor size, high signal on T1WI, Ki-67 expression, nor serum CA-125 level were independent prognostic factors (*p* > 0.05).

**Table 3:** Cox hazard model survival analysis based on clinical and MRI features

	HR	P	95% CI	
Age	2.46	0.04	1.04	5.84
Type II	0.72	0.64	0.18	1.39
CA-125 level (IU/mL)				
< 35	(Reference)			
35–200	1.61	0.57	0.30	8.52
201–500	0.21	0.25	0.01	3.15
> 500	0.35	0.37	0.04	3.53
Ki-67 expression (%)				
< 50	(Reference)			
50–75	0.48	0.28	0.12	1.83
> 75	1.08	0.95	0.10	11.68
FIGO Stage				
I	(Reference)			
II	1.45	0.66	0.27	7.74
III	6.45	0.03	1.25	33.40
IV	23.54	0.01	2.18	254.51
Maximum diameter (mm)	0.98	0.95	0.53	1.81
Component				
Mixed	(Reference)			
Cyst	2.48	0.14	0.75	8.24
Solid	3.69	0.03	1.12	12.15
mean ADC (Log)	2.24	0.25	0.56	8.95
Septa	0.52	0.25	0.17	1.59
High signal on T_1_WI	0.98	0.96	0.39	2.47

Note: Reference groups: ovarian cancer type I, FIGO stag I, mixed components, absence of septa, and absence of T_1_WI high signal. Multiple imputation approach was applied

## Discussion

Ovarian cancer is the most malignant cancer in women’s reproductive diseases, accounting for more than 90% of deaths from ovarian cancers [18]. Type I and Type II OEC resemble two distinct ovarian cancer subtypes based on molecular characteristics [3]. The significance of distinguishing the two OEC subtypes is that Type I has different biological behaviors, treatment responses, and gene mutations from Type II [5], and Type II ovarian cancer (most being high-grade serous ovarian cancer) account for more than 70% of all ovarian cancer deaths [5, 18–20]. Considering the recent treatment strategy advancements [21–23], a minimally invasive approach and fertility-sparing approaches are necessary for patients with BOT tumors and patients of reproductive age with gynecological cancers [24, 25]. From this point of view, accurate preoperative evaluation of ovarian tumors play an important role in setting treatment plans [26].

Our findings indicated that both clinical and MRI features can distinguish between Type I and Type II OEC cancer to some extent. The mean ADC value in Type II was much lower than that in Type I. The old age, an advanced FIGO stage, and a solid component predicted poorer prognosis for this OEC patients group. To the best of our knowledge, no previous studies on the role of MRI features in differentiating Type I and Type II OEC cancers by using a large cohort has been reported.

Our finding that Type I cancer occurred more often in younger age groups than Type II cancer is in accordance with reported literature [19]. This may be explained by the fact that over a half of our patients with Type I cancer had borderline tumors (74/142), which usually occur in young patients. Both Ki-67 expression and CA-125 level were higher in Type II than in the Type I cancer group, because the patients with Type II cancer were hospitalized with a more advanced FIGO stage than those in the Type I group. Similar findings have also been reported in recently published literature [20].

On MRI, tumors with either vegetation or irregularly thickened septa or a wall of more than 3 mm is often suggestive of malignancies [27, 28]. Our study showed that both a thickened septa and high signal on T1WI in the lesion were more often observed in patients with Type I OEC than those with Type II OEC. The possible reason may be that Type II cancer comprised of only one pathological subtype, while there were four subtypes identified in Type I OEC patients resulting in much heterogeneity in the lesion. Also, previous research found that hyperintense cystic components were often detected on T1WI in clear cell and endometroid ovarian cancer than in other subtypes [29, 30]. These findings are supported by our study. Our results showed that the mainly solid tumors only constituted a small part of the total sample in both Type I (21/145) and Type II (15/105) groups. This is different from the study sample of Liu et al. where a solid or predominantly solid mass was detected on MRI in 44 patients with Type II cancer (38.6%) [20].

Our previous studies together with other research declared that ovarian cancer has much lower ADC value than benign disease [7, 8, 31, 32]. However, there is limited literature reporting on the usefulness of ADC measurements in discriminating OEC subtypes. Our study disclosed that the mean ADC value was lower in Type II cancer than in Type I cancer with statistical significance regardless of lesion components. Similar results were reported by Feng et al., who used multiple MRI diffusion models among 32 patients [33]. Another of our findings that a larger tumor was more likely to be found in patients with Type I cancer was also supported by Alcázar et al. and Liu D et al.’s studies [19, 20]. The reason is, as we mentioned above, that BOT was the most common pathological type in the Type I group, usually displaying a relatively large tumor when detected on MRI. However, in this study, when leaving BOTs out, the mean ADC value difference was still observed between Type I and Type II group. In one study with 43 BOTs and 43 OECs, the authors used multiparametric MRI to differentiate BOTs from FIGO stage I OEC by applying logistic regression analysis [34]. They found that mixed components and predominantly solid components, as well as a thickened irregular septa, were more frequently seen in OECs.

Our study revealed significant differences in ADC measurements between clinical and MRI subtype groups. However, no difference was noted in tumor size and treatment response subgroups. That means that tumor size could not influence the ADC value by itself. For the treatment response group, such results need to be confirmed in large cohort samples. Our findings also disclosed that ADC value was inversely related with Ki-67 expression in ovarian cancer tumor tissue, which is in accordance with the previous literature [35, 36]. This character reflects the ovarian tumor cell proliferation to some extent. Notably, this correlation was only observed in the patients with Type I cancer, but not in those with Type II cancer. Lindgren et al. reported that lower ADC values were significantly associated with high Ki-67 expression among 40 patients with primary and metastatic ovarian cancers on a 3.0 T MRI unit [37]. However, they did not describe the details in pathological types as well as OEC subtypes.

In 2015, a comprehensive study with near 2700 OEC patients reported that a significantly increased overall survival for those with Type I tumors was observed compared to those with Type II cancer after 730 days of follow-up [18]. In our study, FIGO stage was an independent prognostic factor that an advanced stage was always associated with poorer prognosis [18]. Our finding that old age predicting a poor prognosis for OEC is in line with another study’s results [38]. However, we did not find any difference in survival time between Type I and Type II OEC group. Clear cell ovarian cancer has the poorest prognosis compared with others and HGSC is the next OEC subtype that has a poor survival outcome. In a recent article, researchers reported that survival for patients with serous and endometrioid ovarian cancer was improved during the past decade; meanwhile, no improvement was seen for those with mucinous and clear cell carcinoma subtypes [38]. Our findings validate that finding to some extent. Chen et al. analyzed 410 OEC patients who had achieved a complete clinical remission [39]. They found that nadir CA-125 level and histotype were independent predictors of progress-free survival (PFS) and overall survival (OS) duration. The PFS and OS durations for patients with Type I OEC were longer than those with Type II OEC [39].

In this study, a solid mass was associated with poor survival in both Type I and Type II cancers. However, the real mechanism is unclear. We also found that a lower ADC value was not associated with worse survival at the end follow-up time point, which differs from Lindgren’s study [37]. The possible explanation is that their study also included metastatic ovarian cancer. We believe the inclusion of metastatic ovarian cancer could significantly affect the results of survival analysis. Future studies with more sample size is needed to testify our result.

This study had several limitations. First, our study was a retrospective research. Some missing data was found during the data collection period, especially patients’ follow-up information. All data was collected in our single institute so that it could partly balance out this bias. Secondly, the ADC value was manually measured on the selected area based on individual habits. Standardization in measurement may influence the final results. In this study, all the ADC measurements were performed by one person in order to minimize such a difference. Thirdly, in these studied samples, in the Type I group, BOTs comprised of more than 50% of the cohort samples (74/145) and future studies may be needed to clarify the true ADC measurement differences between Type II and Type I groups, without including borderline tumors. Besides, sixty-six patients with advanced ovarian cancer in the Type II group often had peritoneal implants and pelvic fluid extension, making primary lesion identification difficult. All of these conditions may eventually effect the ADC measurements in both groups.

## Conclusions

MR features combined with an ADC value are helpful in categorizing OEC subtypes. ADC value can reflect tumor proliferative ability in Type I cancer. Solid mass component on MR imaging could predict poor prognosis for OEC patients.

## Additional file

Additional file 1: **Table S1.** Details of parameters for MRI imaging protocols. **Table S2.** Statistical difference of ADC measurements in various groups based on clinical and MRI features. **Figure S1.** A 66-years old woman with clear cell tumor (Ic). **Figure S2.** Stem-and-Leaf Plots of the calculated ADC values (10^− 3^/mm^2^/s) within between Type I and Type II cancer group. **Figure S3.** Scatter Plots of Ki-67 expression and the mean ADC value in Type II cancer group. (DOCX 472 kb)

## Data Availability

The authors declare that all data supporting the findings of this study are available within the article.

## Abbreviations

ADC: Apparent diffusion coefficient
BOT: Borderline tumor
CA-125: Carbohydrate antigen 125
DWI: Diffusion weighted imaging
FIGO: International Federation of Gynecology and Obstetrics
HIS: Hospital information system
HR: Hazard ratio
OEC: Ovarian epithelial cancer
OR: Odds ratio
PACS: Picture archive communication system
