# Supplementary material for: Magnetic resonance imaging in categorization of ovarian epithelial cancer and survival analysis with focus on apparent diffusion coefficient value: correlation with Ki-67 expression and serum cancer antigen-125 level
Source: J Ovarian Res. 2019 Jun 26;12:59. doi: 10.1186/s13048-019-0534-0 (PMC6595619; doi:10.1186/s13048-019-0534-0)
Supplement: Supplementary file 1 — Table S1. Details of parameters for MRI imaging protocols. Table S2. Statistical difference of ADC measurements in various groups based on clinical and MRI features. Figure S1. A 66-years old woman with clear cell tumor (Ic). Figure S2. Stem-and-Leaf Plots of the calculated ADC values (10− 3/mm2/s) within between Type I and Type II cancer group. Figure S3. Scatter Plots of Ki-67 expression and the mean ADC value in Type II cancer group. (DOCX 472 kb) [file 13048_2019_534_MOESM1_ESM.docx]

**Supplementary Tables**

| **Additional file 1: Table S1.** Details of parameters for MRI imaging protocols. | | | | | | | | | | |  |
| --- | --- | --- | --- | --- | --- | --- | --- | --- | --- | --- | --- |
| Parameters | T1WI | | T2WI | | FS-T2WI | | DWI | | Contrast-enhanced MRI | |  |
| Repetition / echo time (msec) | | 550/10 | | 4000/83 | | 8000/83 | | 2800/81 | | 4.89/2.38 | |
| Echo trains per slice | 44 | | 19 | | 19 | |  | |  | |  |
| Sequence | TSE | | TSE | | TSE | | EP2D | | VIBE | |  |
| Bandwidth(Hz) | 178 | | 260 Hz | | 260 | | 1250 Hz | | 400 Hz | |  |
| Thickness(mm) | 4 | | 4 | | 4 | | 5 | | 3 | |  |
| Field of view (mm) | 350 | | 350 | | 350 | | 300 | | 380 | |  |
| Voxel size(mm) | 1.5×1.1×4.0 | | 1.1×1.1×4.0 | | 1.4×1.4×4.0 | | 2.7×1.9×5.0 | | 1.7×1.2×3.0 | |  |
| Flip angle (degrees) | 150 | | 144 | | 150 | |  | | 10 | |  |

|  |  |  |  |  |  |
| --- | --- | --- | --- | --- | --- |
|  | **Additional file 1: Table S2.** Statistical difference of ADC measurements in various groups based on clinical and MRI features. | | | |  |
|  | Group items | ADC (mean ± S.D.) × 10^-3^s/m^2^ | P value |  |  |
|  | Clinical features |  |  |  |  |
|  | *CA-125 level(U/L)* |  | 0.000 |  |  |
|  | <500 | 1105 ± 475 |  |  |  |
|  | >500 | 851 ± 231 |  |  |  |
|  | *Maximum diameter(cm)* |  | 0.424 |  |  |
|  | <10 | 1009 ± 413 |  |  |  |
|  | >10 | 1096 ± 481 |  |  |  |
|  | *Ki67 expression* |  | 0.000 |  |  |
|  | <50% | 1109 ± 453 |  |  |  |
|  | >50% | 795 ± 234 |  |  |  |
|  | *FIGO* |  | 0.01 |  |  |
|  | I and II | 1059 ± 47.8 |  |  |  |
|  | III and IV | 902 ± 29.3 |  |  |  |
|  | *Relapse or dead* |  | 0.672 |  |  |
|  | yes | 1025 ± 376 |  |  |  |
|  | no | 1056 ± 456 |  |  |  |
|  | MRI features |  |  |  |  |
|  | *Component* |  | 0.000 |  |  |
|  | solid | 1046 ± 444 |  |  |  |
|  | mixed | 1163 ± 490 |  |  |  |
|  | cystic | 840 ± 323 |  |  |  |
|  | *T_1_ high signal* |  | 0.000 |  |  |
|  | present | 1209 ± 437 |  |  |  |
|  | absent | 977 ± 431 |  |  |  |
|  | *Septa* |  |  |  |  |
|  | present | 1213 ± 509 | 0.000 |  |  |
|  | absent | 950 ± 370 |  |  |  |

**Figure legends**

**
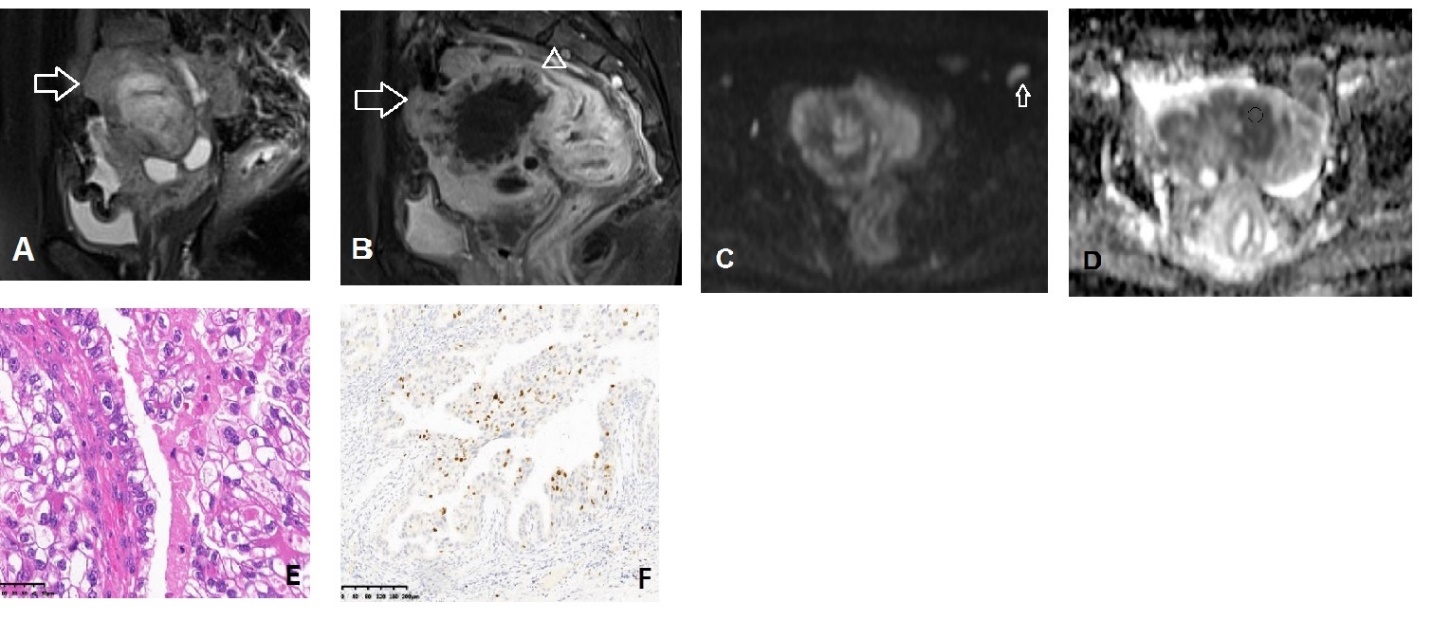
**

**Additional file 1: Figure S1**. A 66-years old woman with clear cell tumor (Ic). On sagittal fs-T2WI(A)，the mainly solid tumor (arrow) centrally occupied the pelvisand the mass showed avid enhancement on post-enhanced images(B) and the mass backwardly invaded the anterior wall of rectum(arrow head); On DWI, the solid tumor showed high signal and the corresponding ADC value (D) was 1134(853-1446) ×10^-3^s/m^2^. Note, the enlarged node (arrow) beside the iliac artery. Haematoxylin and eosin staining of tumor (original magnification × 400, E) and Ki-67 stain picture (original magnification × 100, 5% expression in one view field, F).

**
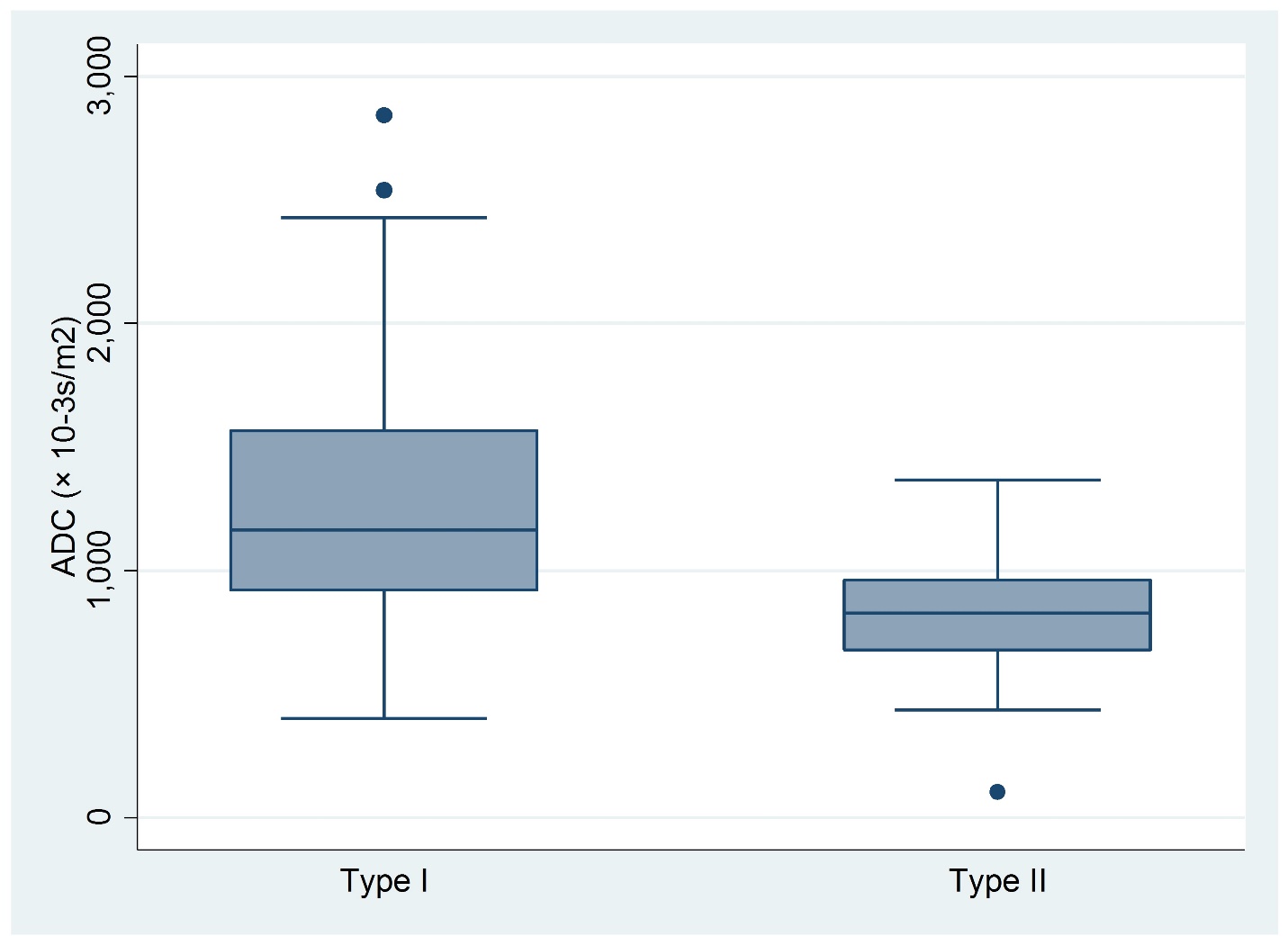
Additional file 1: Figure S2**. Stem-and-Leaf Plots of the calculated ADC values (10^-3^/mm^2^/s) within between Type I and Type II cancer group.

**
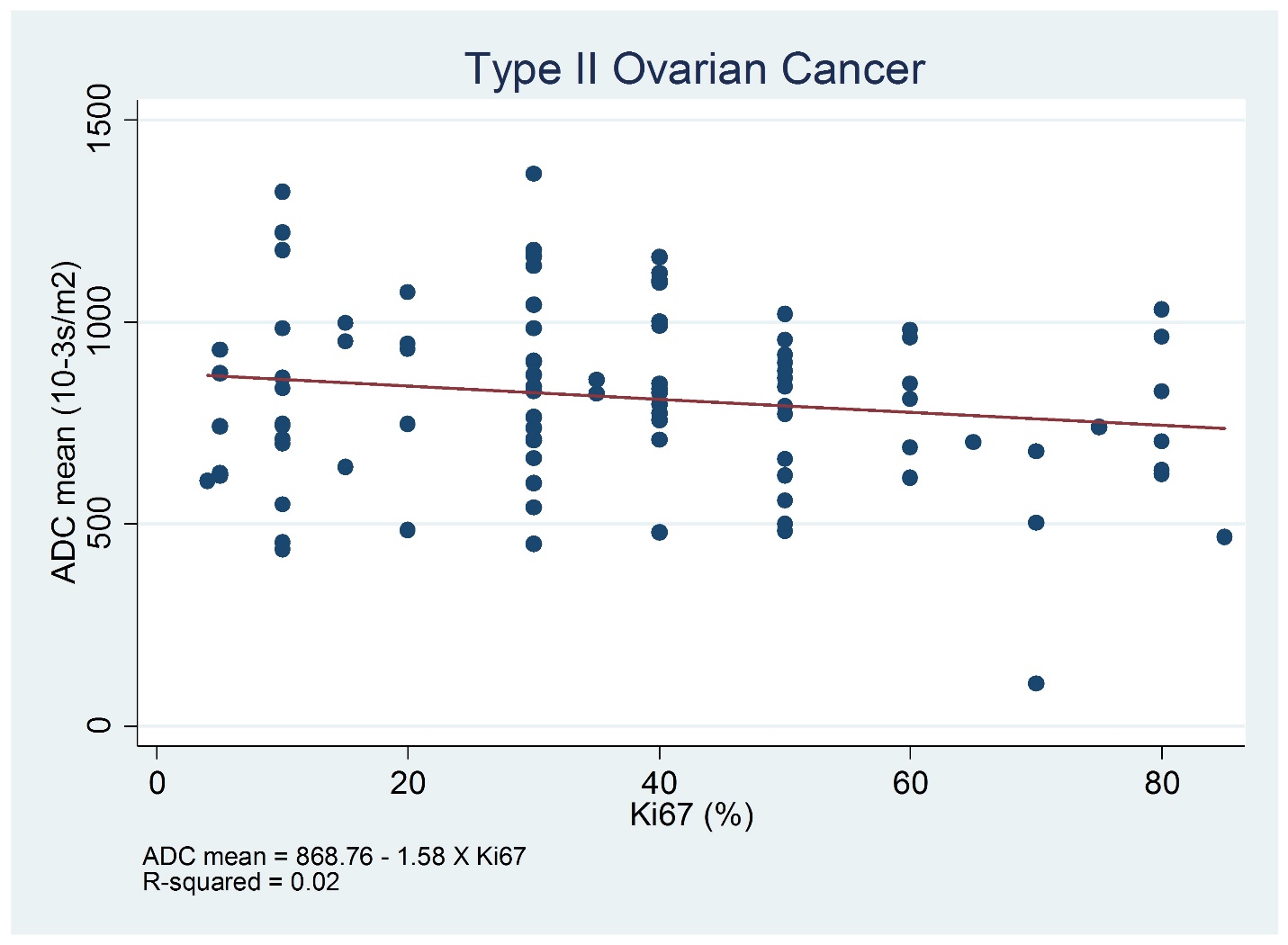
Additional file 1: Figure S3**. Scatter Plots of Ki-67 expression and the mean ADC value in Type II cancer group.
